# Development of an automated closed-loop β-blocker delivery system to stably reduce myocardial oxygen consumption without inducing circulatory collapse in a canine heart failure model: a proof of concept study

**DOI:** 10.1007/s10877-021-00717-w

**Published:** 2021-05-10

**Authors:** Takuya Nishikawa, Kazunori Uemura, Yohsuke Hayama, Toru Kawada, Keita Saku, Masaru Sugimachi

**Affiliations:** Department of Cardiovascular Dynamics, National Cerebral and Cardiovascular Center Research Institute, Kishibe-Shinmachi 6-1, Suita, Japan

**Keywords:** Beta-blocker, Automated drug delivery, Closed-loop control, Hemodynamics

## Abstract

Beta-blockers are well known to reduce myocardial oxygen consumption (MVO_2_) and improve the prognosis of heart failure (HF) patients. However, its negative chronotropic and inotropic effects limit their use in the acute phase of HF due to the risk of circulatory collapse. In this study, as a first step for a safe β-blocker administration strategy, we aimed to develop and evaluate the feasibility of an automated β-blocker administration system. We developed a system to monitor arterial pressure (AP), left atrial pressure (P_LA_), right atrial pressure, and cardiac output. Using negative feedback of hemodynamics, the system controls AP and P_LA_ by administering landiolol (an ultra-short-acting β-blocker), dextran, and furosemide. We applied the system for 60 min to 6 mongrel dogs with rapid pacing-induced HF. In all dogs, the system automatically adjusted the doses of the drugs. Mean AP and mean P_LA_ were controlled within the acceptable ranges (AP within 5 mmHg below target; P_LA_ within 2 mmHg above target) more than 95% of the time. Median absolute performance error was small for AP [median (interquartile range), 3.1% (2.2–3.8)] and P_LA_ [3.6% (2.2–5.7)]. The system decreased MVO_2_ and P_LA_ significantly. We demonstrated the feasibility of an automated β-blocker administration system in a canine model of acute HF. The system controlled AP and P_LA_ to avoid circulatory collapse, and reduced MVO_2_ significantly. As the system can help the management of patients with HF, further validations in larger samples and development for clinical applications are warranted.

**Supplementary Information:**

The online version contains supplementary material available at 10.1007/s10877-021-00717-w.

## Introduction

Beta-blockers are drugs that bind to beta-adrenergic receptors and inhibit the binding of norepinephrine and epinephrine [1]. This inhibition reduces the cardiac workload and myocardial oxygen consumption (MVO_2_) through negative chronotropic and inotropic effects [2, 3]. This is the key mechanism of cardioprotection by β-blockers. Other mechanisms of cardioprotection by β-blockers include the suppression of renin release [4], reduction of myocardial oxygen stress [5], and normalisation of calcium handling in the sarcoplasmic reticulum [6].

In the chronic phase of heart failure (HF), it is well known that β-blockers reduce cardiac remodelling and improve the prognosis of HF patients with reduced ejection fraction [7, 8]. In the acute phase, perioperative stress increases norepinephrine and epinephrine spillover [9] causing tachycardia and hypertension, and increases MVO_2_ [10]. Excessive MVO_2_ leads to myocardial ischemia. The European guideline recommends using β-blockers in the perioperative phase of cardiac surgery [11]. In addition, withdrawal of β-blockers in the acute phase of acute decompensated HF worsens the prognosis [12]. These studies indicate the benefit of β-blockers in acute phase. However, in patients with impaired cardiac function, the negative inotropic and chronotropic effects of β-blockers may lead to circulatory collapse, i.e., cardiogenic shock or pulmonary congestion [13–15]. This risk of circulatory collapse hampers the use of β-blockers in high-risk patients. A safe method of β-blocker administration will permit the use of β-blockers in high-risk patients and may provide cardioprotective effects. Since the range of dosage adjustment is narrow in high-risk patients, we hypothesized that automated drug regulation based on negative feedback of hemodynamics can control the dosage of β-blocker appropriately. We previously developed automated cardiovascular drug delivery systems that administered inotropic agents, vasodilators, diuretics, and/or infused fluids to simultaneously control arterial pressure (AP), cardiac output (CO), and left atrial pressure (P_LA_) under acute HF condition [16–18]. We hypothesize that by allowing a mild drop of AP to an acceptable range, we can extend the system to handle the infusion of β-blockers in acute HF. Jannet et al. [19] reported an automated drug delivery system for β-blockers, which controlled heart rate (HR) by administering esmolol, a short-acting β-blocker. However, since they focused on HR only, the risk of esmolol-induced circulatory collapse remained unsolved.

With this background, we believe that a safe β-blocker administration strategy will provide cardioprotective effects and be useful in the management of patients with acute HF. As a first step for the strategy, we developed an automated β-blocker administration system that administered landiolol, an ultra-short-acting β-blocker, using negative feedback control of hemodynamics. The aim of this study is to prove the concept of our proposed system and to evaluate the feasibility of the system in a canine model of HF.

## Methods

### Automated drug delivery system

**Fig. 1:**
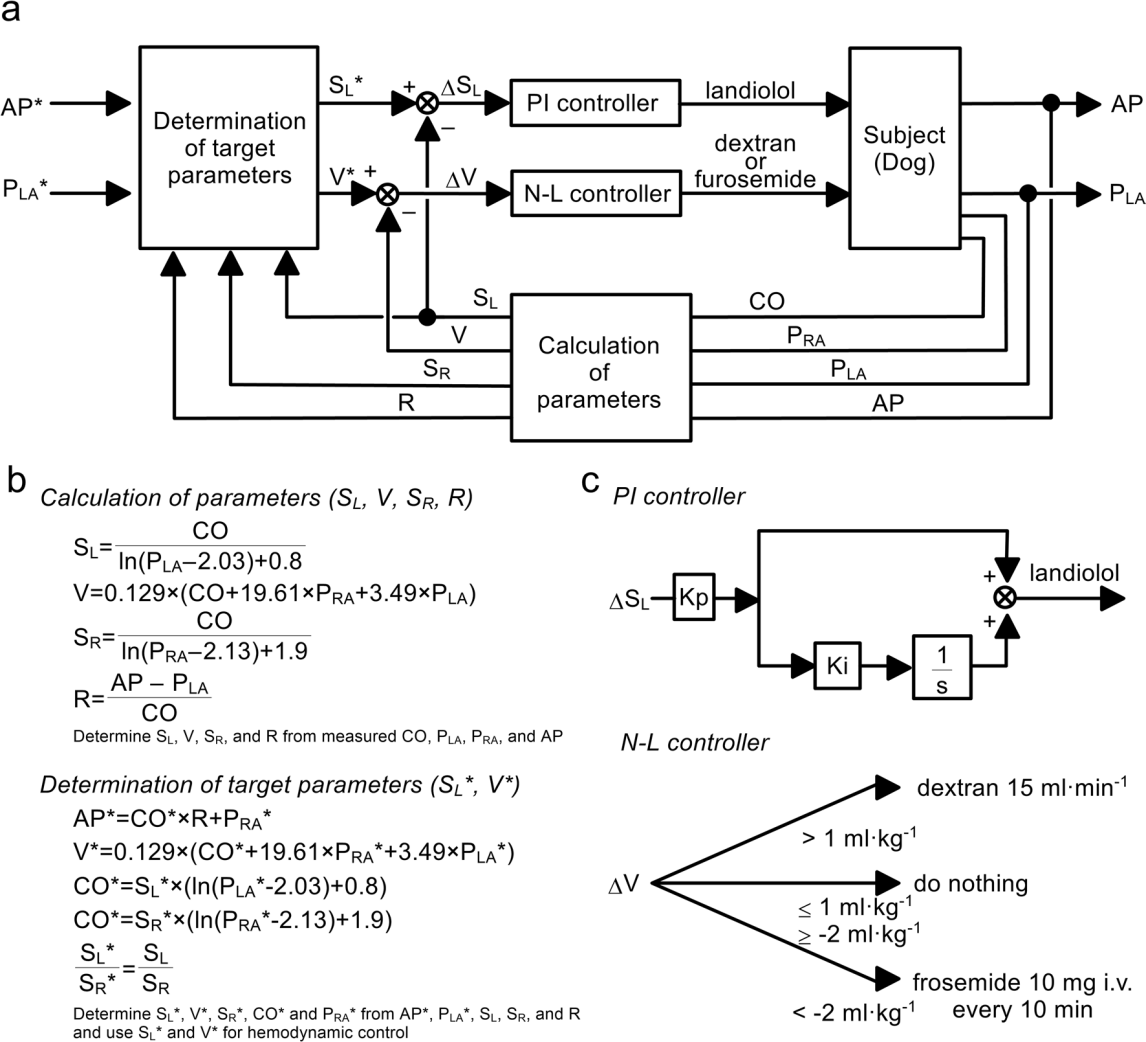
**a** Schematic representation of the automated drug delivery system to control arterial pressure (AP) and left atrial pressure (P_LA_). From measured AP, P_LA_, right atrial pressure (P_RA_) and cardiac output (CO), the system calculates hemodynamic parameters comprising S_L_ (slope of Frank-Starling curve for left ventricle), S_R_ (slope of Frank-Starling curve for right ventricle), stressed blood volume (V) and systemic vascular resistance (R). From S_R_, R, target AP (AP^*^) and target P_LA_ (P_LA_^*^), the system determines target S_L_ (S_L_^*^) and V (V^*^). The infusion rate of landiolol is controlled by a proportional-integral (PI) controller to minimise the difference between S_L_^*^ and S_L_ $(\Delta {\text{S}}_{{\text{L}}} )$. The infusion rate of dextran and injection of furosemide are controlled by a nonlinear (N-L) controller to minimise the difference between V^*^ and V $(\Delta {\text{V}})$. By controlling S_L_ and V, AP and P_LA_ reach preset target values. **b** Equations used in parameter calculation and determination of target parameters. P_RA_^*^ indicates target P_RA_, and S_R_^*^ indicates target S_R_. **c** Details of the controllers of drugs. Kp, proportional gain; Ki, integral gain. *s* is a Laplace operator, and 1/*s* indicates integration

In this study, by extending the systems that we reported previously [16–18], we developed an automated drug delivery system to control the infusion rates of landiolol and dextran, and the injection of furosemide to reduce MVO_2_ without inducing circulatory collapse in subjects with acute HF. Figure 1a shows the scheme of our system. The details of the system are described in Supplemental material. In brief, the user sets the target values of mean AP (AP*) and mean P_LA_ (P_LA_*). The system measures AP, CO, P_LA_ and right atrial pressure (P_RA_). From the measured hemodynamic variables, the system calculates the slope of the Frank-Starling curve for left (S_L_) and right (S_R_) ventricles, systemic vascular resistance (R) and stressed blood volume (V) (Fig. 1b top panel). From AP* and P_LA_*, and the measured hemodynamic data, the system determines the target values of S_L_ (S_L_*) and V (V*) from AP* and P_LA_*, and calculates hemodynamic parameters (Fig. 1b bottom panel). To minimise the difference between S_L_* and S_L_ $(\Delta {\text{S}}_{{\text{L}}} )$, a proportional-integral feedback controller adjusts the infusion rate of landiolol (Fig. 1c top panel). To minimise the difference between V* and V$(\Delta {\text{V}})$, a nonlinear feedback controller adjusts the infusion rate of dextran or the injection of furosemide (Fig. 1c bottom panel). Using these feedback controllers, the system administers landiolol and dextran or furosemide to bring mean AP and mean P_LA_ to the preset target values.

### Animal experiments

#### Rapid pacing-induced heart failure model

We used six adult mongrel dogs weighing 21.0–29.5 kg (male/female, 4/2). We induced anaesthesia with intravenous thiamylal sodium (25 mg kg^−1^), performed endotracheal intubation, and maintained an appropriate anaesthesia level during the experiment by continuous inhalation of isoflurane (1‒2%) under mechanical ventilation. Body temperature was maintained between 37 and 38 °C. We performed transthoracic echocardiography to measure left ventricular end-diastolic dimension (LVDD), left ventricular end-systolic dimension (LVDS) and ejection fraction under normal conditions. After inserting a bipolar pacing lead (Model BT-60P, Star Medical Inc., Tokyo, Japan) to the right ventricular apex through the right jugular vein, we connected a generator (Model SIP-501, Star Medical, Tokyo, Japan) to the pacing lead and implanted it in a subcutaneous pocket at the neck [17]. We closed the incisions and withdrew anaesthesia. One day after implantation, we started rapid ventricular pacing at a rate of 230 beats·min^−1^ and continued for three weeks to induce HF.

#### Experimental preparation and automated drug control

We performed experiments the day after discontinuing rapid pacing. Under anaesthesia induced as described above, we performed transthoracic echocardiography to assess LVDS, LVDD and ejection fraction, and recorded an electrocardiogram (ECG) to calculate HR. We placed 8-Fr sheath introducers in the right femoral artery to measure AP, and in the right and left femoral veins for infusing dextran and landiolol, respectively, and placed a 10-Fr sheath introducer in the right jugular vein to measure P_RA_. We inserted a catheter to the coronary sinus via the right jugular vein under fluoroscopy. After a left thoracotomy and pericardial incision, we introduced a catheter into the left atrial appendage to measure P_LA_. We placed ultrasonic flow probes at the ascending aorta (20PS; Transonic, Ithaca, NY) and the left circumflex artery (2.5PS; Transonic, Ithaca, NY) to measure CO and coronary flow, respectively.

We attached an infusion pump (CFV-3200, Nihon Kohden, Tokyo, Japan) for administering landiolol, and a roller pump (Minipulse 3, Gilson, Middleton, WI) for administering dextran. We controlled these pumps via a laboratory computer (LC-72N10, Logitec, Tokyo, Japan). We used the sheath introducer at the femoral vein for injecting furosemide according to a command signal from the computer. We digitised all hemodynamic data at 200 Hz with an analogue-to-digital converter (AD 12-16, Contec, Osaka, Japan) and stored the data in a dedicated laboratory computer system.

#### Experimental protocol

After stabilisation for 30 min, we connected the closed-loop system to the animal. We set AP* as 10–15 mmHg lower than baseline AP, but not lower than 70 mmHg. We set P_LA_* as baseline P_LA_, but not higher than 18 mmHg. After activating the system by closing the feedback loops of drug administration (Fig. 1), we recorded the infusion rates of landiolol and dextran and the injection of furosemide on the computer. The performance of the system was monitored for 60 min, and arterial and coronary sinus blood samples were collected simultaneously at baseline (0 min), 30, and 60 min after system activation.

After completion of the protocol, the dogs were euthanized with an intravenous injection of pentobarbital and potassium chloride. We measured left ventricular weight after excising the adjacent right ventricular muscle and valvular tissues.

#### Myocardial oxygen consumption and blood gas analysis

We measured oxygen contents of the arterial and coronary sinus blood samples using a co-oximeter (AVOXimeter 4000; Instrumentation Laboratory, Bedford, MA). According to Fick’s principle, the product of coronary flow and the difference between arterial and coronary sinus oxygen contents yields MVO_2_. We normalized MVO_2_ by 100 g left ventricular weight (LVW). We also performed blood gas analysis of arterial blood samples using a blood gas analyser (ABL800 FLEX; Radiometer, Tokyo, Japan) to assess pH, electrolytes, lactate, and partial pressure oxygen and carbon dioxide.

### Data analysis

#### Performance of the automated drug delivery system.

We defined the acceptable range for AP as AP*–5 mmHg or above, and for P_LA_ as P_LA_* + 2 mmHg or below. We evaluated the percentage of time in which mean AP or mean P_LA_ was within the acceptable range. To evaluate the precision and stability of the system, we calculated the performance error (PE), median PE (MDPE), median absolute PE (MDAPE), and wobble by the following equations [20].

$${\text{PE}}\left( {\text{t}} \right) = \frac{{{\text{Variable}}\left( {\text{t}} \right) - {\text{Target}}\left( {\text{t}} \right)}}{{{\text{Variable}}\left( {\text{t}} \right)}} \times 100$$

$${\text{MDPE}} = {\text{median}}\left\{ {{\text{PE}}\left( {\text{t}} \right)} \right\}$$

$${\text{MDAPE}} = {\text{median}}\left\{ {\left| {{\text{PE}}\left( {\text{t}} \right)} \right|} \right\}$$

$${\text{Wobble}} = {\text{median}}\left\{ {\left| {{\text{PE}}\left( {\text{t}} \right) - {\text{MDPE}}} \right|} \right\}$$

where t represents a time unit. Divergence is the slope of the regression line between $\left| {{\text{PE}}\left( {\text{t}} \right)} \right|$ and t (min). MDPE, MDAPE, wobble, and divergence indicate the bias, accuracy, stability, and trend of the absolute error, respectively. Since hemodynamics was stabilised after approximately 15 min, we calculated PE for AP and P_LA_ from 15 to 60 min after the system was activated.

### Statistics

Data are expressed as median (interquartile range). We used Wilcoxon signed rank test to compare echocardiographic variables obtained before and after creating the rapid pacing-induced HF model. We performed a post hoc power analysis to assess the effect size and power of the tests. Wilcoxon signed rank test and Friedman’s test were performed using R version 3.4.3 (R Foundation for Statistical Computing, Vienna, Austria). Post hoc power analysis was performed using G*Power Version 3.1.9.6 (Faul et al. 2007). We considered differences to be significant at *p* < 0.05.

## Results

### Rapid pacing-induced heart failure model

**Fig. 2:**
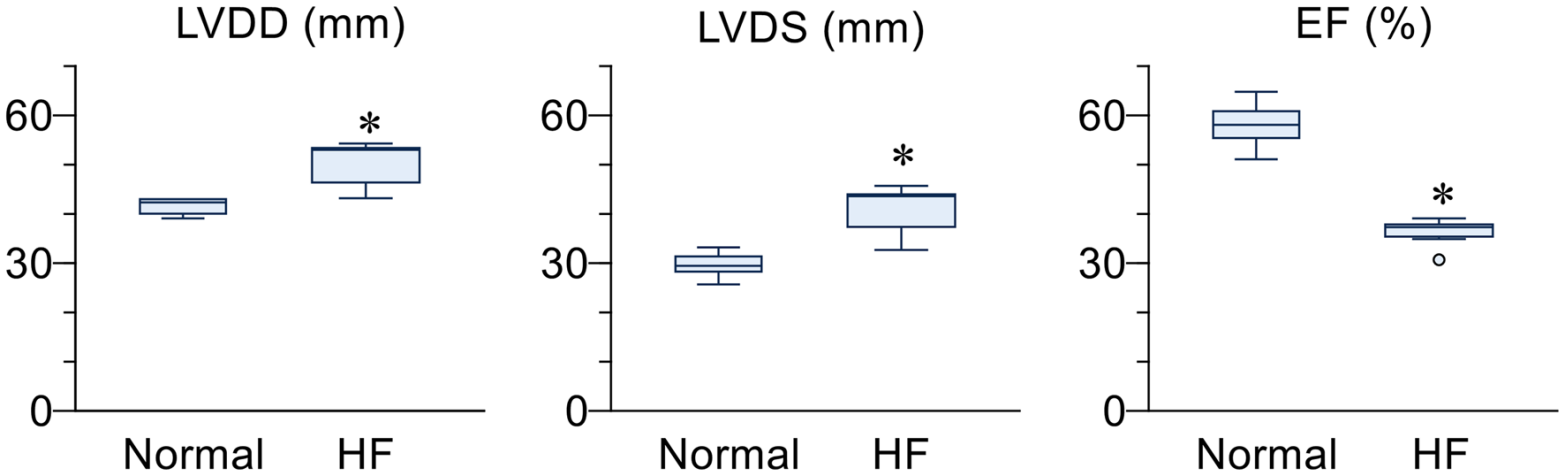
Echocardiographic variables of cardiac function measured before (Normal) and after three weeks of rapid cardiac pacing to induce heart failure (HF). LVDD, left ventricular end-diastolic dimension; LVDS, left ventricular end-systolic dimension; EF, ejection fraction. *p < 0.05 vs. normal

Rapid pacing significantly increased LVDD [before to after 3-week pacing: 42 (40–43) to 53 mm (46–53), *p* < 0.05] and LVDS [29 (28–31) to 44 mm (37–44), *p* < 0.05], and reduced ejection fraction [58 (55–61) to 37 mm (35–38), *p* < 0.05] (Fig. 2). Mean AP and mean P_LA_ just before activation of the drug delivery system were 87 mmHg (85–89) and 17 mmHg (15–19), respectively. These data indicated that rapid pacing for three weeks induced HF in the animals.

### Hemodynamic control by the automated drug delivery system

**Fig. 3:**
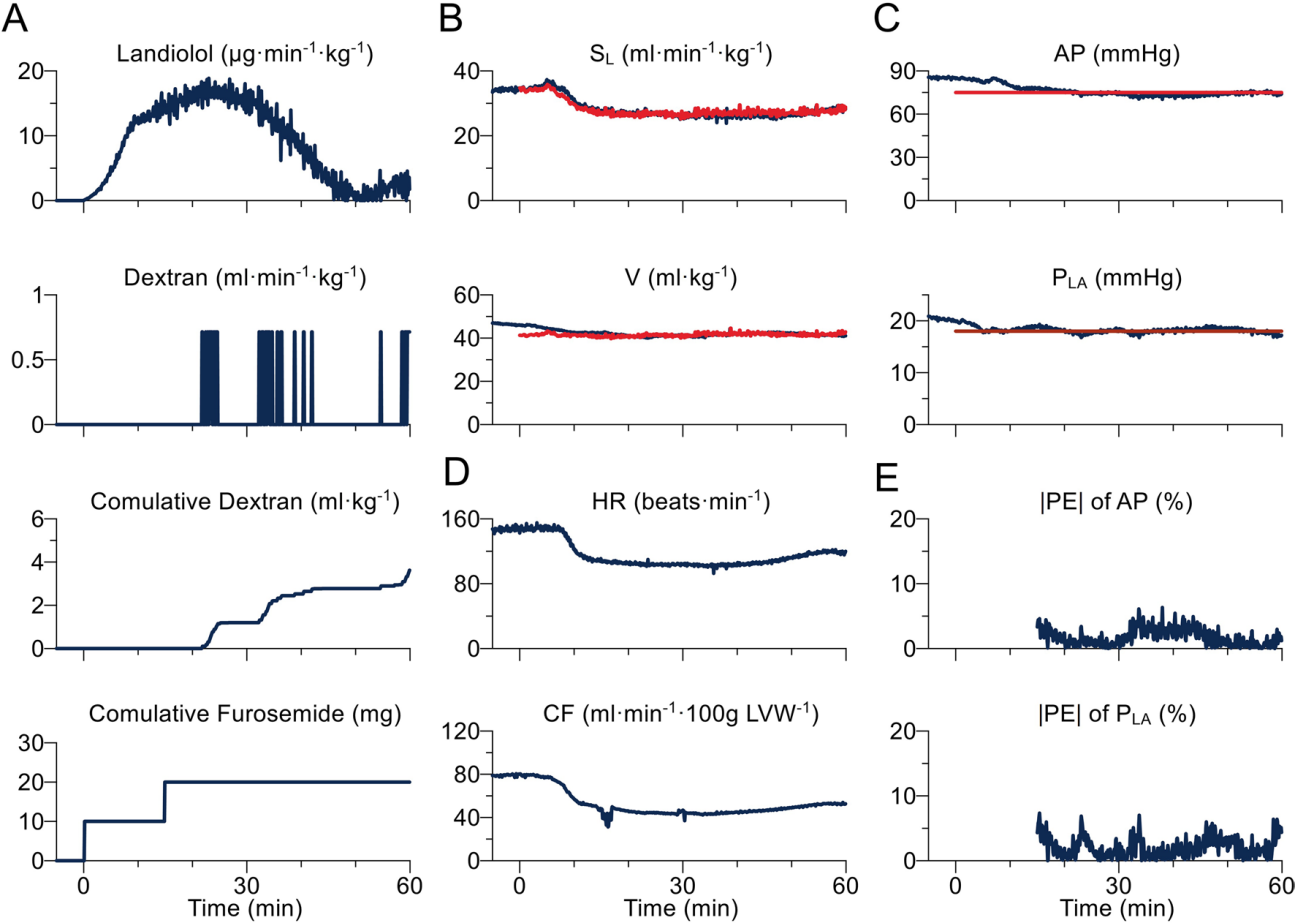
Representative time series data of one dog during hemodynamic control by the automated drug delivery system. **a** Infusion rates of landiolol and dextran, and cumulative doses of dextran and furosemide; **b** slope of Frank-Starling curve for left ventricle (S_L_) and stressed blood volume (V); **c** mean arterial pressure (AP) and mean left atrial pressure (P_LA_); **d** heart rate (HR) and coronary flow (CF); **e** absolute performance error (|PE|) for AP and P_LA_. Navy blue and red lines indicate measured and target values, respectively

Figure 3 shows the representative time series data of one dog during hemodynamic control by the system. After activation at 0 min, the system started to control the infusion rates of landiolol and dextran, and injection of furosemide (Fig. 3a). As a result, the hemodynamic parameters S_L_ and V approached their respective target values (Fig. 3b). By controlling S_L_ and V, mean AP and mean P_LA_ reached the preset target values accurately (Fig. 3c). HR and coronary flow were reduced markedly within 20 min (Fig. 3d). From 15 to 60 min after the system activation, |PE| for mean AP or mean P_LA_ was less than 10%.

**Fig. 4:**
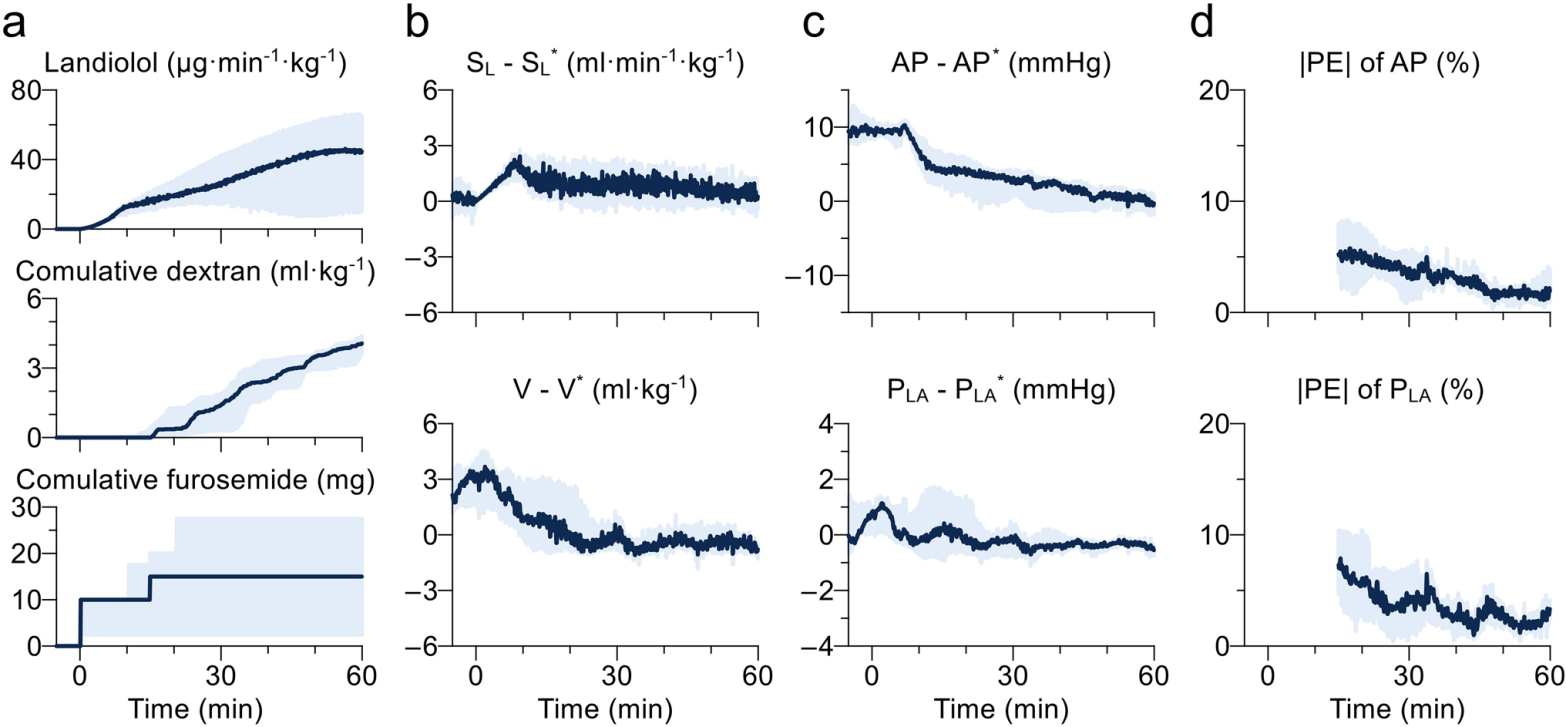
Summarised time series data of six dogs during hemodynamic control by the automated drug delivery system. Data are expressed as median (solid line) and interquartile range (blue area). **a** Infusion rates of landiolol and dextran, and cumulative doses of dextran and furosemide; **b** difference between measured and target values of slope of Frank-Starling curve for left ventricle (S_L_) and stressed blood volume (V); **c** difference between measured and target values of mean arterial pressure (AP) and mean left atrial pressure (P_LA_); **d** absolute performance error (|PE|) for AP and P_LA_

Figure 4 summarises the results of time series data of all six dogs. The solid line and the grey shade indicate median and interquartile range, respectively. Figure 4a shows the infusion rate of landiolol and cumulative doses of dextran and furosemide. The trend of landiolol infusion rate varied among the animals. In three dogs, the infusion rate of landiolol initially increased, then gradually decreased. In the other three dogs, the infusion rate of landiolol increased gradually over time during hemodynamic control. In all dogs, the infusion rate of landiolol averaged over the 60-min period was 26.2 μg min^−1^ kg^−1^ (11.4–37.9), and the cumulative dose of dextran was 4.1 ml kg^−1^ (3.7–4.4). Furosemide was injected in three dogs, and the cumulative furosemide dose was 15 mg (2.5–27.5). Figure 4b, c show the errors of S_L_, V, AP, and P_LA_. |PE| for mean AP or mean P_LA_ was less than 10% from 15 to 60 min after system activation (Fig. 4d).

**Fig. 5:**
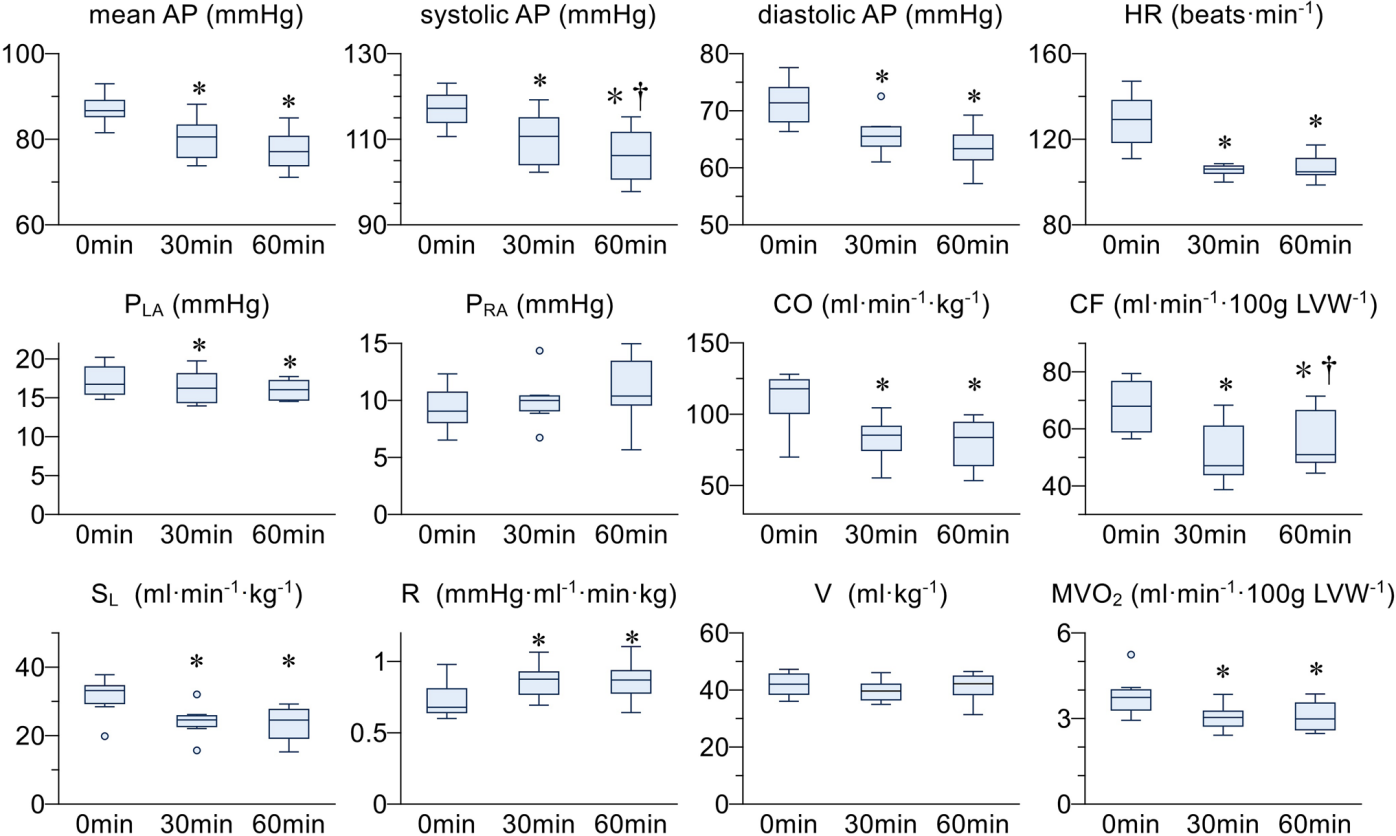
Summarised hemodynamic and energetics data of six dogs obtained at 0 min, 30 min, and 60 min after the system was activated. Boxes represent median and interquartile range. Whiskers represent minimum and maximum values. Outliers are represented as circles. AP, arterial pressure; P_LA_, left atrial pressure; P_RA_, right atrial pressure; HR, heart rate; CO, cardiac output; CF, coronary flow; S_L_, slope of Frank-Starling curve for left ventricle; R, systemic vascular resistance; V, stressed blood volume; MVO_2_, cardiac oxygen consumption; *p < 0.05 vs. 0 min, **p < 0.05 vs. 30 min

Figure 5 summarises the hemodynamic data and MVO_2_ at baseline (0 min), 30, and 60 min after system activation. Mean AP [baseline: 87 (85–89); 30 min: 81 (76–83); 60 min: 77 mmHg (74–81), *p* < 0.01], systolic AP [baseline: 117 (114–120); 30 min: 111 (104–115); 60 min: 106 mmHg (101–112), *p* < 0.01] and diastolic AP [baseline: 71 (68–74); 30 min: 66 (64–67); 60 min: 63 mmHg (61–66), *p* < 0.01] decreased significantly at 30 and 60 min compared to baseline. However, in all dogs, the system maintained mean AP not lower than 70 mmHg, and systolic AP not lower than 100 mmHg during the 60-min hemodynamic control period. Mean P_LA_ was slightly but significantly lower compared to baseline [baseline: 16.7 (15.5–19.0); 30 min: 16.2 (14.4–18.1); 60 min: 16.0 mmHg (14.7–17.2), *p* < 0.05], whereas P_RA_ remained unaltered [baseline: 9.1 (8.1–10.7); 30 min: 10.0 (9.1–10.4); 60 min: 10.4 mmHg (9.6–13.4), *p* = 0.3]. These results indicated that our system prevented cardiogenic shock and/or pulmonary congestion. MVO_2_ decreased significantly at 30 and 60 min compared to baseline [baseline: 3.8 (3.4–4.0); 30 min: 2.9 (2.7–3.2); 60 min: 3.0 ml min^−1^ (2.6–3.4)·100 g LVW^−1^, *p* < 0.01]. HR [baseline: 129 (118–138); 30 min: 106 (104–107); 60 min: 105 bpm (103–111), *p* < 0.01], CO [baseline: 118 (101–124); 30 min: 86 (75–92); 60 min: 84 ml min kg^−1^ (64–95), *p* < 0.01], and coronary flow [baseline: 68 (59–77); 30 min: 51 ml min^−1^ (48–66) 100 g LVW^−1^, *p* < 0.01] decreased significantly at 30 and 60 min compared to baseline.

### Performance of the automated system

Table 1 shows the performance of the automated system. In all dogs, mean AP was controlled within the acceptable range 100% of the time. Mean P_LA_ was controlled within the acceptable range 99.7% (94–100) of the time. These results indicated that the system did not induce circulatory collapse during almost the entire period of hemodynamic control. The MDAPE (%) for mean AP and mean P_LA_ was 3.1 (2.2–3.8) and 3.6 (2.2–5.7), respectively; MDPE (%) was 3.1 (− 0.2–3.7) and − 1.8 (− 3.6 to − 0.04); wobble (%) was 2.1 (1.6–2.5) and 2.4 (1.1–3.0); and divergence (% min^−1^) was -0.09 (− 0.13 to − 0.02) and − 0.09 (− 0.17 to − 0.01). These results indicated that the system automatically controlled AP and P_LA_.

**Table 1:** Performance of the automated drug control system

AP (mmHg)					
Animal number	Time in acceptable range^a^ (%)	MDAPE (%)	MDPE (%)	Wobble (%)	Divergence (% min^−1^)
1	100	3.8	3.7	3.7	− 0.25
2	100	9.4	9.4	1.7	− 0.11
3	100	1.5	− 1.1	2.5	− 0.05
4	100	2.1	− 2.1	1.0	− 0.1
5	100	3.7	3.7	2.6	− 0.14
6	100	2.5	2.5	1.6	− 0.08
	100 (100–100)	3.1 (2.2–3.8)	3.1 (− 0.2–3.7)	2.1 (1.6–2.5)	− 0.1 (− 0.12–0.05)

P_LA_ (mmHg)					
Animal number	Time in acceptable range^b^ (%)	MDAPE (%)	MDPE (%)	Wobble (%)	Divergence (%·min^−1^)
1	99	3.2	− 2.2	3.0	− 0.24
2	100	6.5	− 6.5	1.7	0.18
3	92	1.8	0.5	2.5	− 0.004
4	100	1.5	− 1.5	1.0	− 0.003
5	81	6.2	2.6	2.6	− 0.5
6	100	4.1	− 4.1	1.6	0.03
	99.7 (94–100)	6.5 (2.2–5.6)	− 1.8 (− 3.6–0.03)	2.4 (1.1–3.0)	− 0.003 (− 0.18–0.02)

Bottom rows show median (interquartile range) of 6 dogs

*AP* arterial pressure, *MDAPE* median absolute performance error, *MDPE* median performance error, *P*_*LA*_ left atrial pressure

^a^Mean AP within 5 mmHg below target

^b^Mean P_LA_ within 2 mm above target

### Blood gas analysis

Table 2 shows the data of blood gas analysis. Despite the significant reduction in CO, lactate remained unaltered. This result suggested that the system maintained peripheral perfusion adequately. All other blood gas parameters did not change significantly during hemodynamic control.

**Table 2:** Results of blood gas analysis of six dogs

	0 min		30 min		60 min		p-value
pH	7.45	(7.42–7.46)	7.45	(7.43–7.46)	7.45	(7.41–7.45)	0.68
PaO_2_ (mmHg)	99	(94–125)	86	(72–118)	107	(84–118)	0.61
PaCO_2_ (mmHg)	28.4	(26.5–29.3)	27.5	(26.6–28.5)	28	(27.2–29.2)	0.88
HCO_3_^−^ (mmol l^−1^)	18.5	(18.2–18.8)	18.6	(18.2–18.6)	18.4	(18.2–18.7)	0.88
BE (mmol l^−1^)	− 4.7	(− 5.4 to − 3.8)	− 4.6	(− 5.4 to − 4.1)	− 4.7	(− 5.4 to − 4.4)	0.69
Lactate (mg dl^−1^)	11	(9–15)	13	(10–16)	13	(9–15)	0.68

Data are expressed as median (interquartile range). 0 min, 30 min, and 60 min represent the time after activating the automated drug delivery system

*PaO*_*2*_ partial pressure of arterial oxygen, *PaCO*_*2*_, partial pressure of arterial carbon dioxide, *BE* base excess

### Post hoc power analysis

Table S1 in Supplemental material summarises the results of a post hoc power analysis. Although Friedman’s test showed no significant difference in P_RA_, stressed blood volume (V), pH, partial pressure of arterial oxygen (PaO_2_), partial pressure of arterial carbon dioxide (PaCO_2_), bicarbonate (HCO_3_^−^), base excess (BE), and lactate by hemodynamic control, all these variables showed small (< 0.3) effect sizes and low (< 0.8) statistical power (1-β).

## Discussions

Beta-blockers are well known for their efficacy in HF, but their use in the acute phase of HF is limited due to the risk of circulatory collapse. As a first step to safely administer β-blockers for cardioprotection in the acute phase of HF, we have developed and evaluated the feasibility of an automated drug administration system. The system administered the ultra-short-acting beta-blocker, landiolol, to decrease MVO_2_ while controlling mean AP and mean P_LA_ to preset target values in a canine model of HF. It is the first system that automatically adjusts β-blockers to achieve cardioprotection while maintaining hemodynamics. In order to validate the utility and efficacy of the system for acute cardiovascular care, further studies with large sample sizes and development of clinically applicable system are needed.

The system controls the infusion of landiolol and dextran as well as injection of furosemide based on negative feedback control of hemodynamics. Mean AP and mean P_LA_ reached the preset target values within 15 min after the system was activated. During the duration of hemodynamic control by the system, although AP decreased from baseline, mean AP and systolic AP were maintained higher than 70 and 100 mmHg, respectively. Furthermore, the system decreased HR, P_LA_ and MVO_2_ significantly. Although the system reduced CO significantly, it did not alter lactate level. Therefore, our system administered landiolol and reduced MVO_2_ without inducing circulatory collapse under acute HF condition. In this study, we proved the concept of an automated drug administration system and evaluated its feasibility. Further studies are needed to demonstrate the superiority of this concept over existing therapies and its long-term prognostic effect.

### Comparison to other automated drug delivery systems

Recently, various automated drug delivery systems have been developed in the field of anaesthesia [21–23] and for the control of volume status [24], AP [25–27] and HR [19]. Especially, the development of automated anaesthesia systems is progressing; for example, closed-loop feedback control of the bispectral index for stable control of hypnosis. Several meta-analyses have shown that automated anaesthesia delivery can be more effective than manually controlled anaesthesia by anaesthesiologists in attaining tight control with a specified range of target variables [28–30]. These systems also succeed to reduce the doses of anaesthetics delivered and shorten the recovery time. Regarding the use of β-blockers, Jannet et al. [19] proposed a closed-loop control system for esmolol infusion. They designed the system to control the infusion rate of esmolol so as to bring the ventricular rate to preset target value in dogs with induced atrial fibrillation. Negative feedback control of esmolol infusion rate stably controlled the ventricular rate. However, since they did not consider other hemodynamic parameters, AP and P_LA_ during esmolol infusion could not be predicted or controlled.

To control AP and P_LA_ during landiolol infusion, we designed the present system by extending our previous automated drug delivery systems based on the circulatory equilibrium framework [16–18]. In this framework, circulatory equilibrium (CO, P_LA_, and P_RA_) is determined by the intersection of the Frank-Starling curves and the venous return surface [31–33]. By controlling the Frank-Starling curves with dobutamine and the venous return surface with dextran and furosemide, the previous systems control CO, P_LA_, and P_RA_ to desired values. Based on the same framework but with different logic, the present system controls the Frank-Starling curves with landiolol and the venous return surface with dextran or furosemide to bring mean AP and mean P_LA_ to pre-set target values (see Supplemental Material). In the canine HF model, the system automatically controlled mean AP and mean P_LA_. Percent time in acceptable range for both AP and P_LA_ was high, similar to previous reports for other closed-loop control systems [20, 23], indicating that the system avoided circulatory collapse (low AP and/or high P_LA_). Furthermore, the control performance indices (MDPE, MDAPE, wobble, and divergence) were small, comparable to those observed in previous reports of closed-loop hemodynamic control systems [26, 34]. These results suggest that our system using β-blockers achieves the same degree of hemodynamic control as previously reported closed-loop systems.

Regarding the control speed, the system stabilized the hemodynamics within 15 min. This is considerably shorter than the clinical time required for dose titration of landiolol (more than 30 min) [14]. Therefore, the present system is the first system that achieves automated control of hemodynamics using β-blockers while decreasing MVO_2_ in dogs with HF.

### Acute hemodynamic effects of the system

We designed the present system to infuse a maximum dose of landiolol in individual subject, since the benefit of β-blockers depends on the dosage. In anaesthetized dogs, Satoh et al. [35] reported that β-blockers dose-dependently reduced AP, HR and MVO_2_.

However, a high dose of β-blocker suppresses cardiac pumping function, especially under HF conditions. Indeed, infusion of landiolol by our system decreased AP and CO. Although there is no clear clinical evidence of acceptable AP range under acute HF condition, previous studies recommended to maintain mean AP of over 65 mmHg in cardiogenic shock [36] or following septic shock [37]. Therefore, we set AP* as 10–15 mmHg lower than mean baseline AP, but not lower than 70 mmHg. During hemodynamic control by our system, mean AP decreased from 87 mmHg (85–89) at baseline to 77 mmHg (73–81) at 60 min, and the minimum AP was 70 mmHg. Previous clinical studies evaluating the efficacy of landiolol to treat atrial fibrillation or ventricular tachy-arrhythmia in acute HF have indicated that landiolol induces hypotension in 7.5–20% of HF patients [14, 15]. On the other hand, although systolic AP was not a controlled variable in our system, it was maintained at not lower than 100 mmHg in all animals in this study. Hence, our system did not induce critical hypotension and achieved safe control of hemodynamics using β-blocker. Regarding CO, Cooper et al. [38] reported that CO observed after acute phase treatment in patients with acute decompensated HF was not associated with mortality or cardiovascular hospitalisation, whereas pulmonary capillary wedge pressure was a strong predictor of these events. Therefore, we designed the system to control AP and P_LA_, but not CO. Although CO decreased from 118 ml min^−1^ kg^−1^ (101–124) at baseline to 84 ml min^−1^ kg^−1^ (64–95) at 60 min, blood lactate level remained unaltered. This result suggests that the decrease of CO (approximately 30% reduction) when using the system may not reach a critical level. Another possibility is that landiolol may reduce peripheral oxygen consumption, thereby improving the oxygen demand‒supply balance. Indeed, several previous results suggest that β-blockers suppress systemic oxygen utilisation or renal oxygen consumption [39, 40]. However, the post hoc power analysis showed low statistical power for blood lactate level, indicating that the sample size (n = 6) was not large enough to conclude that blood lactate level was not elevated. In addition, blood lactate level alone is not sufficient to assess the damage to peripheral organs (including brain, kidney, and liver). Biomarkers such as serum creatinine, cystatin C, and hepatic transaminases need to be evaluated. However, hemodynamic control was tested for only 60 min in this study, which was not long enough to evaluate such biomarkers. Further studies with larger sample size and more detailed evaluation of organ damage are needed.

### Clinical application of our system

Since we invasively measured CO, P_RA_ and P_LA_ in this study, the present experimental setting may not apply directly to clinical practice. In the clinical setting, pulmonary artery catheterization (PAC) allows measurements of continuous thermodilution CO, central venous pressure, and diastolic pulmonary arterial pressure. By estimating P_LA_ from diastolic pulmonary arterial pressure, the system can be applied clinically. However, PAC is invasive and sampling for continuous thermodilution CO requires one minute. Thus, we have proposed less invasive monitoring methods for CO and P_LA_ using the ultrasound technique [17, 41, 42]. In brief, CO is estimated by left ventricular outflow tract velocity time integral and AP. P_RA_ is substituted by the jugular vein pressure (P_JV_). P_LA_ is estimated from the velocity of mitral and tricuspid annulus and P_JV_. These monitoring methods may be selected based on the needs for PAC. The use of these monitoring methods should be explored to further develop the present system for clinical application.

### Limitations

The limitations of this study have to be addressed. First, the sample size was small. The low statistical power shown in the post hoc power analysis (Table S1, Supplemental material) indicated that the sample size (n = 6) was not large enough to rule out type 2 error in several variables. Second, all animals were anesthetized and ventilated. Anaesthesia and ventilation are known to affect hemodynamics [43]. The results of this study may be different under awake conditions. However, we consider that the negative feedback mechanism used in the present system may compensate for the variations in drug responses between anesthetized and awake states. Third, we only assessed acute MVO_2_ change. Further studies are needed to elucidate whether the improved myocardial energetics achieved by the present system ameliorates myocardial damage and improves long-term survival in HF subjects. Fourth, we used a rapid pacing-induced HF model. It is well known that the cardiac function in this model improves spontaneously once rapid pacing is stopped [44]. To observe whether cardiac function improved spontaneously, we monitored hemodynamics in three of six dogs for 120 min after the end of hemodynamic control (Table S2 in Supplemental material). In these three dogs, we observed no systematic improvement of hemodynamics. These results may imply that cardiac function did not recover spontaneously during 60 min of hemodynamic control. However, a control group without β-blocker administration is required to exclude the effect of spontaneous cardiac recovery for accurate interpretation of the results. Finally, no comparison was made with conventional use of landiolol. Further studies are needed to compare with fixed landiolol or manual control of landiolol to demonstrate its superiority over current treatments.

## Conclusions

We have developed and evaluated the feasibility Queryof an automated drug delivery system that adjusts the infusion rate of landiolol to reduce MVO_2_ without inducing circulatory collapse. In a canine model of HF, this system significantly reduced MVO_2_ indicating the potential of cardioprotection. The system controlled both mean AP and mean P_LA_ within the acceptable control ranges more than 95% of the time, and MDPAE for AP and P_LA_ was less than 10%. These results indicate that the system may improve cardiac energetics and reduce myocardial damage in acute HF. This study is the first step to develop a method of safe administration of β-blockers for cardioprotection in the acute phase of HF. To establish this system for clinical use, further validation studies with large sample sizes and development for clinical applicable system are needed.

## Supplementary Information

Below is the link to the electronic supplementary material.

Supplementary file1 (DOCX 45 kb)

## Data Availability

The datasets used and/or analysed during this study are available from the corresponding author on reasonable request.

## Code availability

The code used in this study are available from the corresponding author on reasonable request.
