# Supplementary material for: Development of an automated closed-loop β-blocker delivery system to stably reduce myocardial oxygen consumption without inducing circulatory collapse in a canine heart failure model: a proof of concept study
Source: J Clin Monit Comput. 2021 May 10;36(3):849–60. doi: 10.1007/s10877-021-00717-w (PMC9162998; doi:10.1007/s10877-021-00717-w)
Supplement: Supplementary file 1 — Supplementary file1 (DOCX 45 kb) [file 10877_2021_717_MOESM1_ESM.docx]

**Development of an automated closed-loop β-blocker delivery system to stably reduce myocardial oxygen consumption without inducing circulatory collapse in a canine heart failure model: a proof of concept study.**

*Journal of Clinical Monitoring and Computing*

**Supplement material**

**Authors:**

Takuya Nishikawa, Kazunori Uemura, Yohsuke Hayama, Toru Kawada, Keita Saku,

and Masaru Sugimachi

**Corresponding author:**

Takuya Nishikawa, MD, PhD

Department of Cardiovascular Dynamics

National Cerebral and Cardiovascular Center

Suita 564-8565, Japan

TEL +81-6-6170-1070

FAX +81-6-6170-1752

E-mail: nishikawa.takuya75@ncvc.go.jp

**Automated drug delivery system**

We developed an automated drug delivery system that administers landiolol, dextran and furosemide based on negative feedback control of hemodynamics. The scheme of the system is described in the main manuscript. First, the user sets the target values of arterial pressure (AP^*^) and left atrial pressure (P_LA_^*^). The system continuously measures the subject’s arterial pressure (AP), left atrial pressure (P_LA_), right atrial pressure (P_RA_), and cardiac output (CO) and then low-pass filtered these hemodynamic variables at a cut-off frequency of 0.1 Hz to calculate mean values to be used in the closed-loop feedback system. Based on the circulatory equilibrium framework [1–3], the system calculates the subject’s hemodynamic parameters comprising systemic vascular resistance (R), stressed blood volume (V) and the slopes of the Flank-Starling curves for the left ventricle (S_L_) and right ventricle (S_R_) from the measured hemodynamic variables using the following equations.

$$\begin{aligned} R=\frac{AP-P_{\mathrm{RA}}}{\mathrm{CO}}\#\left( 1 \right) \end{aligned}$$

$$\begin{aligned} V=0.129\times\left( CO+19.61\times P_{\mathrm{RA}}+3.49\times P_{\mathrm{LA}} \right)\#\left( 2 \right) \end{aligned}$$

$$\begin{aligned} S_{L}=\frac{\mathrm{CO}}{\ln\left( P_{\mathrm{LA}}-2.03 \right)+0.8}\#\left( 3 \right) \end{aligned}$$

$$\begin{aligned} S_{R}=\frac{\mathrm{CO}}{\ln\left( P_{\mathrm{RA}}-2.13 \right)+1.9}\#\left( 4 \right) \end{aligned}$$

From the hemodynamic parameters of AP*, and P_LA_*, the system calculates target S_L_ (S_L_*) and target V (V*) by the algorithm shown below. Assuming that R and the ratio of S_L_ to S_R_ are constant, we can describe target CO (CO*), target P_RA_ (P_RA_*), target V (V*), target S_L_ (S_L_*), target S_R_ (S_R_*) by the following equations.

$$\begin{aligned} \mathrm{AP}^{*}=\mathrm{CO}^{*}\times R+P_{\mathrm{RA}}^{*}\#\left( 5 \right) \end{aligned}$$

$$\begin{aligned} V^{*}=0.129\times\left( \mathrm{CO}^{*}+19.61\times P_{\mathrm{RA}}^{*}+3.49\times P_{\mathrm{LA}}^{*} \right)\#\left( 6 \right) \end{aligned}$$

$$\begin{aligned} \mathrm{CO}^{*}=S_{L}^{*}\left( \ln\left( P_{\mathrm{LA}}^{*}-2.03 \right)+0.8 \right)\#\left( 7 \right) \end{aligned}$$

$$\begin{aligned} \mathrm{CO}^{*}=S_{R}^{*}\left( \ln\left( P_{\mathrm{RA}}^{*}-2.13 \right)+1.9 \right)\#\left( 8 \right) \end{aligned}$$

$$\begin{aligned} \frac{S_{L}^{*}}{S_{R}^{*}}=\frac{S_{L}}{S_{R}}\#\left( 9 \right) \end{aligned}$$

By solving equations (5)－(9), the system determines CO*, P_RA_*, V*, S_L_*, and S_R_* from S_L_, S_R_, R, AP*, and P_LA_*. The system repeats these calculations to determine S_L_* and V* every 10 s.

To minimize the difference between S_L_* and S_L_, a proportional-integral (PI) feedback controller determines the infusion rate of landiolol by the following equations.

$$\begin{aligned} u\left( t \right)=Kp\left( e\left( t \right)+Ki\int_{0}^{t} e\left( \tau\right)d\tau\right)\#\left( 10 \right) \end{aligned}$$

where t is the time increment, u(t) is the infusion rate of landiolol, e(t) is the error signal (S_L_*-S_L_), Kp is the proportional gain, and Ki is the integral gain. We determine the parameters of the PI controller (Kp = -1.5 (unitless), Ki = 0.01 s^-1^) based on the step response of landiolol (data not shown). When u(t) is negative, the system stops landiolol infusion and keeps the integral term constant until u(t) becomes positive.

To minimize the difference between V* and V, a nonlinear feedback controller determines the infusion rate of dextran or injection of furosemide by the following “if-then” rule.

If V* - V > 1 ml·kg^-1^, then infuse dextran 15 ml·min^-1^

If V* - V < -2 ml·kg^-1^, then inject furosemide 10 mg

After furosemide injection, the system temporarily suspends the injection of furosemide for 10 min.

References

1. Uemura K, Kamiya A, Hidaka I, Kawada T, Shimizu S, Shishido T, et al. Automated drug delivery system to control systemic arterial pressure, cardiac output, and left heart filling pressure in acute decompensated heart failure. J Appl Physiol [Internet]. 2006;100:1278–86.

2. Uemura K, Sugimachi M, Kawada T, Kamiya A, Jin Y, Kashihara K, et al. A novel framework of circulatory equilibrium. Am J Physiol Heart Circ Physiol [Internet]. 2004;286:H2376-85.

3. Uemura K, Kawada T, Kamiya A, Aiba T, Hidaka I, Sunagawa K, et al. Prediction of circulatory equilibrium in response to changes in stressed blood volume. Am J Physiol Heart Circ Physiol. 2005;289:H301–7.

**Table S1. Results of post hoc power analyses**

| Variable analyzed by Wilcoxon signed rank test | Effect size dz | Power (1-β) |
| --- | --- | --- |
| LVDD (mm) | 1.58 | 1.0 |
| LVDS (mm) | 1.74 | 1.0 |
| EF (%) | 3.24 | 1.0 |
|  |  |  |
| Variable analyzed by Friedman’s test | Effect size f | Power (1-β) |
| mean AP (mmHg) | 0.93 | 1.0 |
| systolic AP (mmHg) | 0.79 | 1.0 |
| diastolic AP (mmHg) | 0.91 | 1.0 |
| HR (beats min) | 1.82 | 1.0 |
| P_LA_ (mmHg) | 0.26 | 0.6 |
| P_RA_ (mmHg) | 0.28 | 0.7 |
| CO (ml·min·kg^-1^) | 0.79 | 1.0 |
| CF (ml·min^-1^·100g LVW^-1^) | 0.71 | 1.0 |
| S_L_ (ml·min·kg^-1^) | 0.71 | 1.0 |
| R (mmHg·ml^-1^·min^-1^·kg) | 0.47 | 1.0 |
| V (mmHg·ml^-1^) | 0.22 | 0.5 |
| MVO_2_ (ml·min^-1^·100g LVW^-1^) | 0.84 | 1.0 |
| pH | 0.06 | 0.1 |
| PaO_2_ (mmHg) | 0.09 | 0.1 |
| PaCO_2_ (mmHg) | 0.07 | 0.1 |
| HCO_3_^-^ (mmol·l^-1^) | 0.17 | 0.2 |
| BE (mmol·l^-1^) | 0.11 | 0.1 |
| Lactate (mg·dl^-1^) | 0.13 | 0.2 |

Effect size dz and f indicate Cohen’s dz and f, respectively. β indicates probability of type II error. LVDD, left ventricular end-diastolic dimension; LVSD, left ventricular end-systolic dimension; EF, ejection fraction; AP, arterial pressure; HR, heart rate; P_LA_, left atrial pressure; P_RA_, right atrial pressure; CO, cardiac output; CF, coronary artery flow; S_L_, the slope of Frank-Starling curve for left ventricle; R, systemic vascular resistance; V, stressed blood volume; MVO_2_, cardiac oxygen consumption; PaO_2_, partial pressure of arterial oxygen; PaCO_2_, partial pressure of arterial carbon dioxide; BE, base excess.

**Table S2. Hemodynamics at baseline and 120 min after the cessation of hemodynamic control in three dogs**

| Animal | 2 | | 3 | | 4 | |
| --- | --- | --- | --- | --- | --- | --- |
|  | baseline | 120 min after experiment | baseline | 120 min after experiment | baseline | 120 min after experiment |
| HR (bpm) | 125 | 134 | 147 | 152 | 116 | 122 |
| AP (mmHg) | 93 | 127 | 85 | 94 | 82 | 83 |
| P_LA_ (mmHg) | 15.4 | 16.9 | 20.2 | 23.8 | 17.9 | 18.8 |
| P_RA_ (mmHg) | 11.1 | 9.2 | 9.7 | 11.6 | 12.3 | 16.7 |
| CO (ml·min^-1^·kg^-1^) | 128 | 142 | 126 | 161 | 70 | 51 |

The animal numbers correspond to those indicated in Table 1 in the main text. HR, heart rate; AP, arterial pressure; P_LA_, left atrial pressure; P_RA_, right atrial pressure; CO, cardiac output.
